# Serum Levels of IFABP2 and Differences in *Lactobacillus* and *Porphyromonas gingivalis* Abundance on Gut Microbiota Are Associated with Poor Therapeutic Response in Rheumatoid Arthritis: A Pilot Study

**DOI:** 10.3390/ijms24031958

**Published:** 2023-01-19

**Authors:** Oscar Zaragoza-García, Natividad Castro-Alarcón, Gloria Pérez-Rubio, Ramcés Falfán-Valencia, Olivia Briceño, José Eduardo Navarro-Zarza, Isela Parra-Rojas, Mario Tello, Iris Paola Guzmán-Guzmán

**Affiliations:** 1 Faculty of Chemical-Biological Sciences, Autonomous University of Guerrero, Chilpancingo 39087, Mexico; 2 HLA Laboratory, National Institute of Respiratory Diseases Ismael Cosío Villegas, Mexico City 14080, Mexico; 3 Infectious Diseases Research Center, National Institute of Respiratory Diseases Ismael Cosío Villegas, Mexico City 14080, Mexico; 4 Department of Medical Internal, General Hospital Raymundo Abarca Alarcón, Chilpancingo 39019, Mexico; 5 Bacterial Metagenomics Laboratory, Faculty of Chemistry and Biology, University of Santiago of Chile, Santiago 8320000, Chile

**Keywords:** gut microbiota, intestinal fatty-acid binding protein 2, intestinal permeability, therapy, non-response, rheumatoid arthritis

## Abstract

Intestinal dysbiosis is related to the physiopathology and clinical manifestation of rheumatoid arthritis (RA) and the response to pharmacologic treatment. The objectives of this study were (1) to analyze the effect of conventional synthetic disease modifying anti-rheumatic drugs (csDMARDs) on the abundance of gut microbiota’s bacteria; (2) to evaluate the relationship between the differences in microbial abundance with the serum levels of intestinal fatty-acid binding protein 2 (IFABP2), cytokines, and the response phenotype to csDMARDs therapy in RA. A cross-sectional study was conducted on 23 women diagnosed with RA. The abundance of bacteria in gut microbiota was determined with qPCR. The ELISA technique determined serum levels of IFABP2, TNF-α, IL-10, and IL-17A. We found that the accumulated dose of methotrexate or prednisone is negatively associated with the abundance of *Lactobacillus* but positively associated with the abundance of *Bacteroides fragilis*. The *Lactobacillus/Porphyromonas gingivalis* ratio was associated with the Disease Activity Score-28 for RA with Erythrocyte Sedimentation Rate (DAS28-ESR) (r = 0.778, *p* = 0.030) and with the levels of IL-17A (r = 0.785, *p* = 0.027) in the group treated with csDMARD. Moreover, a relation between the serum levels of IFABP2 and TNF-α (r = 0.593, *p* = 0.035) was observed in the group treated with csDMARD. The serum levels of IFABP2 were higher in patients with secondary non-response to csDMARDs therapy. In conclusion, our results suggest that the ratios of gut microbiota’s bacteria and intestinal permeability seems to establish the preamble for therapeutic secondary non-response in RA.

## 1. Introduction

The intestinal dysbiosis related to the presence of bacteria, such as *Prevotella copri* [1,2], *Lactobacillus* [3], and *Collinsella* [4], is associated with the emergence of the clinical manifestation of autoimmunity in rheumatoid arthritis (RA). Gut microbiota is considered a key factor in RA’s physiopathology [5]. Among the mechanisms that explain that association, the citrullination of peptides that favors the production of antibodies against citrullinated proteins produced by bacteria, such as *Porphyromonas gingivalis* and *Aggregatibacter actinomycetemcomitans*, has been showed [6,7]. Another proposed mechanism is the immunoregulation by cytokine production, which has a key role in autoimmunity processes [2,8]. In RA patients, it has been shown that intestinal dysbiosis is associated with the levels of acute-phase reactants, Th1/Th17 cytokines, and with the disease’s clinical activity score [9,10,11,12,13,14,15]. These antecedents suggests that intestinal dysbiosis is related to the clinical diversification of RA [13,16]. 

On the other hand, it has been shown that the presence of some bacterial lineages of gut microbiota has a key role in the clinical status of RA due to their metabolites. These are related to high levels of chemokine’s and IL-17A and to the increased intestinal permeability associated with the decrease of the expression of protein zonula occludens-1 (ZO-1) [4], in which the translocation of bacterial components happens [17,18,19]. In RA patients, elevated lipopolysaccharide (LPS) serum levels, lipopolysaccharide-binding protein (LBP), and soluble CD14 (sCD14) have been reported, which suggests translocation of microbial products from the intestine [18,19]. Tajik et al. [20] reported that the serum levels of proteins such as ZO-1, occludin-1, and claudin are elevated in the early stages of RA and established RA while the intestinal fatty-acid binding protein 2 (IFABP2) serum levels increased in RA patients without pharmacologic treatment and have been related to the clinical activity [18].

Currently, there is a particular interest in studying the mechanisms through which intestinal dysbiosis relates to the metabolism of the conventional synthetic disease modifying anti-rheumatic drugs (csDMARDs) and to the therapeutic response phenotype [21,22]. This is because RA patients with a response phenotype present a higher abundance of the phylum *Bacteroidetes* while in those non-response to treatment present an increase of phyla *Firmicutes* and *Proteobacteria* [23,24,25]. The effect of drugs in RA treatment on the abundance and/or changes in the proportions of intestinal bacteria remains largely unknown, as well as the role that they could have in intestinal permeability and diversification of the treatment response. Matei et al. [18] reported that the levels of LPS and LBP decrease in RA patients that are responsive to biologic DMARD. Similarly, Audo et al. [19] reported that the levels of LBP and sCD14 decrease in patients who respond to DMARDs and that the variations in these markers are associated with the changes in the Disease Activity Score-28 (DAS28). In the present study, we hypothesized that the non-response to RA therapy could be associated with microbiota composition changes and with intestinal permeability. Therefore, this study has the following objectives: (1) to analyze the effect of csDMARDs on differences in the abundance of gut microbiota’s bacteria; (2) to analyze the relation between the abundance of intestinal bacteria and clinical activity markers, and the serum levels of IFABP2, TNF-α, IL-10, and IL-17A, and the response phenotype to csDMARDs treatment in RA.

## 2. Results

### 2.1. Demographic Data, Markers for Inflammation, Intestinal Bacteria Abundance, and Phenotype of Response to Therapy 

The median age of the population was 45 ± 11 years old. The RA patients with csDMARDs treatment presented significantly longer evolution times of the disease and a lower DAS28-ESR when compared to the patients, defined as new-onset RA (NORA) and untreated (*p* = 0.002). The serum levels of rheumatoid factor (RF), TNF-α, IL-10, and IL-17A were similar among the treated patients and the NORA group. However, the IFABP2 serum levels were higher in the NORA group (*p* = 0.035) compared to patients with treatment. On the other hand, even though the abundance of *Lactobacillus* tended to be higher in the NORA group, the differences were not significant. The abundance of *Bacteroides fragilis, Prevotella, Porphyromonas gingivalis*, and *Escherichia coli* were also not different between the studied groups (Table 1). According to the treated patients’ clinical evolution, only 23.08% presented a response phenotype, whereas 46.15% presented a primary non-response phenotype, and 30.77% were classified as with secondary non-response to therapy. 

### 2.2. Relation between Gut Microbiota’s Bacteria Abundance with the Dose of Drugs and Inflammation Markers

The average prescribed dose of methotrexate (MTX) in treated patients varied between 10 and 25 mg per week and for prednisone (PDN) between 2.5 and 10 mg per day. The accumulated dose of MTX to the top dose (25 mg per week) determines an accumulated dose of 500 mg in 6 months of treatment and 1000 mg to the 12 months, approximately. Similarly, in the case of PDN, considering a daily dose of 10 mg, an accumulated dose was estimated within 6 months of therapy of around 1500 mg. In a correlation analysis, it was observed that the abundance of *Lactobacillus* tends towards a negative relation with the accumulated dose of MTX (r = −0.421, *p* = 0.151) (Supplementary Figure S1A) and PDN (r = −0.443, *p* = 0.198) (Supplementary Figure S2A) while the abundance of *Bacteroides fragilis* related positively with the accumulated dose of PDN (r = 0.632, *p* = 0.055); however, these correlations were not statistically significant (Supplementary Figure S2B). In addition, we did not find a significant relation between *Prevotella*, *Porphyromonas gingivalis*, or *Escherichia coli* abundance and the accumulated dose of MTX (Supplementary Figure S1C–E) and PDN (Supplementary Figure S2C–E).

**Figure 1:**
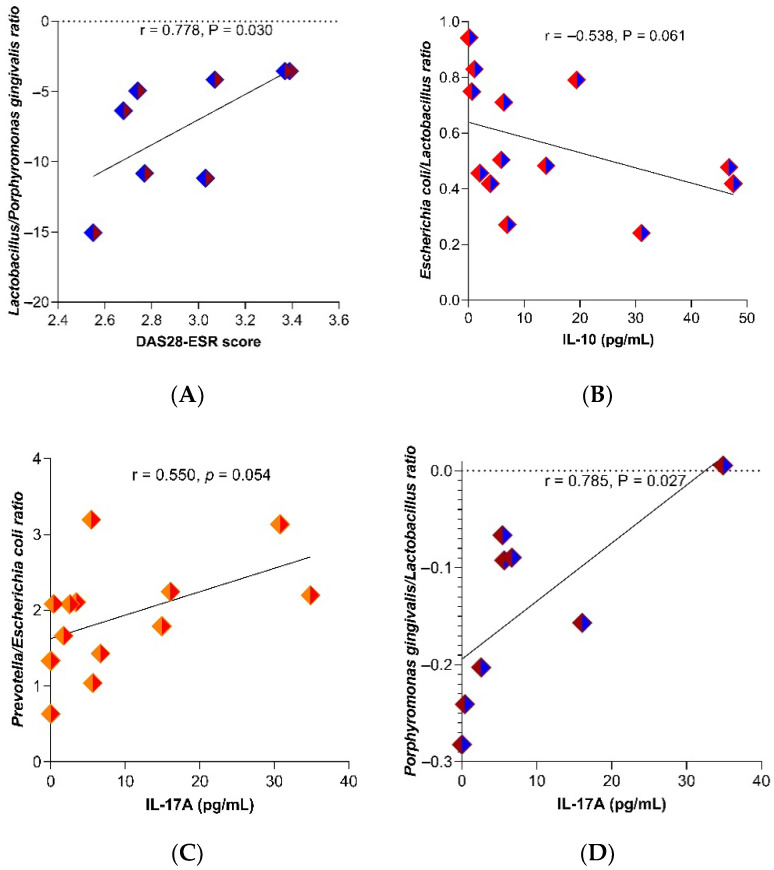
Correlation between clinical activity and cytokines on gut microbiota in RA patients with treated. (**A**) DAS28-ESR, (**B**) IL-10, (**C**,**D**) IL-17A. Spearman’s correlation. r = rho, correlation coefficient. *p*-Value < 0.05 was considered statistically significant.

When we analyzed the relation between the studied bacteria’s abundance, as well as between bacterial ratios, we found that, particularly, the abundance of *Porphyromonas gingivalis* was positively associated with IL-10 serum levels (r = 0.904, *p* = 0.002) and IL-17A (r = 0.761, *p* = 0.028), but not with RF serum levels (r = −0.02, *p* = 0.943). *Porphyromonas gingivalis* was found in 13 out of 23 analyzed samples (5 in the NORA group and 8 in the group with csDMARD). Meanwhile, the *Lactobacillus*/*Porphyromonas gingivalis* ratio was positively associated with the DAS28-ESR (r = 0.778, *p* = 0.030) (Figure 1A). The *Escherichia coli/Lactobacillus* ratio was negatively associated with IL-10 serum levels (r = −0.538, *p* = 0.061), although not statistically significant (Figure 1B), and the *Prevotella*/*Escherichia coli* ratio (r = 0.550, *p* = 0.054) (Figure 1C) and *Porphyromonas gingivalis*/*Lactobacillus* ratio (r = 0.785, *p* = 0.027) were positively associated with the IL-17A serum levels (Figure 1D). 

### 2.3. Relationship of IFABP2 Serum Levels and Inflammation Markers with Gut Microbiota’s Bacteria and Therapy Response Phenotype in RA

**Figure 2:**
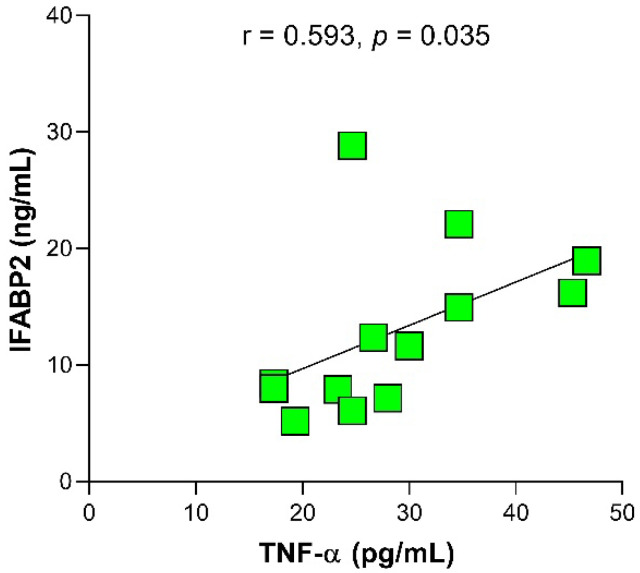
Correlation between seric levels of IFABP2 and TNF-α in RA patients with treatment. Spearman’s correlation. r = rho, correlation coefficient. *p*-Value < 0.05 was considered statistically significant.

Even though the levels of IFABP2 were significantly higher in the NORA group, we found that in the group of patients under csDMARD treatment the IFABP2 serum levels correlated positively with the TNF-α levels (r = 0.593, *p* = 0.035) (Figure 2). 

**Figure 3:**
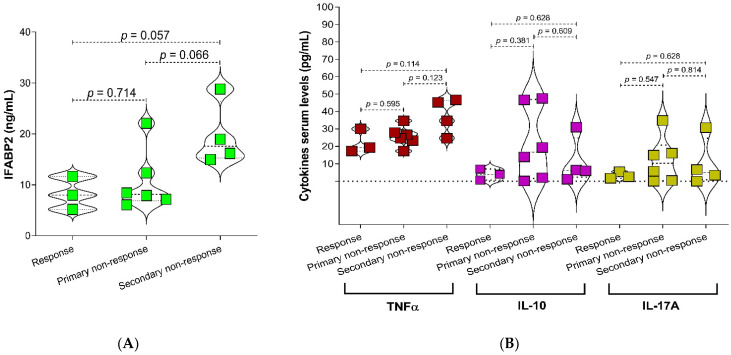
Association of serum levels of IFABP2 and cytokines according to clinical phenotype in RA patients with treatment. (**A**) IFABP2, (**B**) Cytokines (TNF-α, IL-10, and IL-17A). Statistical analyses were performed by Mann-Whitney U test. *p*-Value < 0.05 was considered statistically significant.

Additionally, when comparing the IFABP2 levels between phenotypes of response to therapy, it was found that IFABP2 levels were higher in the group of patients with secondary non-response to therapy (*p* = 0.057) (Figure 3A), where also the TNF-α serum levels showed a tendency to increase, although not statistically significant (Figure 3B). A slight increase was also observed in the abundance of *Prevotella* and *Escherichia coli* in the secondary non-responder group of patients (Supplementary Figure S3). 

## 3. Discussion

The main findings in this study showed that (1) the accumulated dose of MTX and PDN favor the increase of *Bacteroides fragilis*, (2) the changes in proportion of *Lactobacillus*/*Porphyromonas gingivalis* are associated with the increase serum levels of IL-17A and with the clinical activity of the RA, and (3) IFABP2 serum levels are positively associated with TNF-α levels and with secondary non-response to csDMARD in RA. The main strength of this study is that it analyzes the relation between bacteria’s abundance, as well the bacterial ratios with different serological markers related to inflammation in AR; furthermore, this study shows evidence related to the abundance of the gut microbiota’s bacteria and treatment dosage, suggesting a role of treatment on microbiota composition. Moreover, our results suggest that an early change in gut microbiota and intestinal permeability establishes the preamble for the phenotype of response to therapy.

The treatment with corticosteroids (Cs) diminishes the mucus secretion in the colon and promotes changes in the bacterial structure of gut microbiota [26]. In an in vivo study in MRL/1pr mice, it was observed that PDN favors the decrease of bacterial species such as *Mucispirillum*, *Oscillospira*, *Bilophila*, and *Rikenella* [27]. In this study, we observed that the accumulated doses of MTX and PDN within 6 months, approximately, and within 12 months of treatment are associated with the abundance of *Lactobacillus* and *Bacteroides fragilis*; therefore, early changes could potentially establish the dynamics of the primary or secondary non-response to treatment with DMARDs. Similarly to what we report in our study, there is evidence on C57B1/6 mice model that shows that the exposition to Cs is related to the reduction of *Lactobacillaceae* and proposes that the changes in the commensal bacteria could mediate the drug’s anti-inflammatory effect [28]. Previous studies reported a higher abundance of the family *Lactobacillaceae* in subjects in pre-clinical stages of RA [11,29,30,31] compared to the abundance reported in patients recently diagnosed with RA [32] and early RA patients [30]. Therefore, it has been suggested that the changes in quantity of *Lactobacillus* could be an important factor related to the development and progression of RA [3]. 

In this study, we observed that the abundance of *Lactobacillus* was higher in the NORA group and that this abundance decreases in relation to the accumulated dose of MTX and PDN while the abundance of *Bacteroides fragilis* increases. Opposing evidence was reported in a Balb/c model of rats in which the treatment with MTX was seen to diminish the abundance of the species *Bacteroides fragilis* [33] but not that of the order *Lactobacillales* in C57BL/6J rats [34]. However, it has been shown that particularly *Lactobacillus casei* [35] can metabolize MTX just as *Pseudomonas* [36] and the families *Prevotellaceae* and *Anaeroplasmataceae* [37]. Nayack et al. [23] proved in an in vitro model the impact of MTX on *Bacteroidetes*; moreover, they estimated that the concentration of MTX in the proximal gastrointestinal tract (22–220 μM or 10–100 μg/mL) would be sufficient to inhibit the microbial growth [25]. These changes in microbial abundance could be related to the sensitivity to the administered drug’s or the dosage and to the intrinsic capacity of bacteria to metabolize some of the drugs’ structural components.

Interestingly, even though we did not observe a relation between the bacteria’s abundance in their individual form, we found that the variability of *Lactobacillus*, regarding other studied bacteria, showed an association with the clinical activity and inflammation markers. Specifically, we observed that the *Lactobacillus*/*Porphyromonas gingivalis* ratio is positively related to the DAS28-ESR while the *Porphyromonas gingivalis*/*Lactobacillus* ratio is related to the serum levels of IL-17A. These findings are of great interest given that it has been found that *Lactobacillus salivarius* [10] and *Porphyromonas gingivalis* are related to the clinical activity in RA patients [38]. Therefore, the analysis of the changes in the ratios among gut microbiota’s bacteria could potentially reveal their role in RA’s physiopathology.

*Porphyromonas gingivalis* is associated with the production of citrullinated proteins and with the development of RA [6], as well as the expansion of Th17 cells and the signaling of IL-17’s receptor A, which promotes the infiltration of neutrophils in the joints, and the increase of IL-17 and TNF-α [39]. Meanwhile, in vitro, it was found that *Porphyromonas gingivalis* could polarize a cellular response towards a Th17/IL-17 profile by being recognized by the toll-like receptor 2 (TLR2) of antigen-presenting cells and mediating the synthesis of IL-1, IL-6, IL-17, as well as the increase of osteoclastogenesis [40]. Similarly, Marchesan et al. [41] reported in an in vivo model of arthritic rats that *Porphyromonas gingivalis* modulates the levels of IL-1β, IL-6, IL-22, IL-23, TGF-β, and TNF-α. 

In this study, we did not evaluate antibodies to citrullinated protein antigens (ACPAs) levels, but we determined RF serum levels and evaluated their relationship with *Porphyromonas gingivalis* abundance, finding a null relationship. Similar to our results, Scher et al. [42] reported that the presence of *Porphyromonas gingivalis* DNA is not directly related to the presence or levels of ACPAs, nor to RF, thus hypothesizing that this relationship may be subject to interaction with other factors. In that sense, Torato et al. [43] reported that patients with RA positives to HLA-DRB1*04 present higher levels of *Porphyromonas gingivalis* DNA in peripheral blood and synovial tissue, so the genetic factor may be a determinant in the establishment of the host-pathogen relationship.

Furthermore, in our study, the *Prevotella*/*Escherichia coli* ratio was positively related to IL-17A serum levels. Maeda et al. [2] reported that the ribosomal protein L23A from *Prevotella copri* is related to dendritic cell stimulation and IL-17 synthesis. Likewise, IgA antibodies against *Prevotella copri* have been correlated with the serum Th1/Th17 cytokines profiles [44]. In addition, we found that the *Escherichia coli*/*Lactobacillus* ratio was negatively related to IL-10 serum levels. Neuman et al. [9] reported a positive relation between *Escherichia coli* and ESR levels. In this context, the change in the abundance of *Lactobacillus* and other gut microbiota’s bacteria could be an action modulating mechanism of the drugs and inflammation process.

In this study, the IFABP2 serum levels were not associated with clinical characteristics and gut microbiota’s bacteria ratios. However, our study found a relation between the levels of TNF-α and IFABP2 in patients with RA under csDMARD therapy, as well as a relation between IFABP2 and the secondary non-response to therapy. Similarly, Matei et al. [18] observed in RA patients treated with etanercept (anti-TNF) that the IFABP2 serum levels remain elevated in patients with a clinical non-response phenotype. Interestingly, in active Crohn’s disease, it was observed that the infliximab (anti-TNF) treatment could modulate the levels IFABP2 and TNF-α and achieve remission [45]. This evidence supports the findings in this study where IFABP2 exhibited a relation with the non-response phenotype to csDMARD treatment. Currently, the therapy optimization in RA recommend treat-to-target strategies for management and tapering on clinical activity using csDMARD, short term Cs, or targeted therapies with anti-TNFs and biosimilar DMARDs [46,47]. Hence, it is important to evaluate the relationship between IFABP2 and the lack response to others therapeutic schemes in RA.

IFABP2 is a cytosolic protein found mainly in the enterocytes [48], and its elevated serum levels represent a marker for intestinal lesions [49,50]. The relation between TNF-α and IFABP2 allows correlating the chronic inflammation processes and the increase of intestinal permeability. In an in vitro model, it was identified that the pathway of NF-κB has a key role in the activation of TNF-α, whose increment has been related to the decrease of the expression of molecules like ZO-1 and the increase of intestinal permeability [51]. In addition, it has been recognized that NF-κB promotes the expression and enzymatic activity of myosin light chain kinase (MLCK), thus modulating the pathways where this kinase is involved [52]. MLCK has been involved in the paracellular regulation of tight junctions [53], and the evidence indicates that this could be modulated by cytokines such as IL-1β [52], IFN-γ, and TNF-α [54,55]. 

Martinsson et al. [56] reported that short-chain fatty acids derived from bacterial metabolism decrease during the clinical progression of RA. Moreover, it has been demonstrated that butyric acid, oleic acid, and phosphatidylcholine, as well as leptin and TNF-α regulate the expression of IFABP in the cells’ cytosol [57]. TNF-α is a molecule that is closely related to the clinical activity and response phenotype to DMARDs therapy in RA [58,59]. Therefore, the changes in the ratios of gut microbiota, along with the subsequent effect over the production or metabolism of fatty acids, could contribute synergistically to the destabilization of the gut barrier, promoting chronic inflammation and the lack of response to pharmacologic treatment in RA. 

Conversely, the use of prebiotics and probiotics has a more significant presence in the treatment of RA as a strategy to promote the restoration of the commensal microbiota and to decrease the disease’s progression [60], it could also be a future key factor to improve the response to this therapy. This is a pilot study in woman AR patients, and among the limitations of our work, we can consider the lack of male participants; therefore, the results should be interpreted with caution because they are necessary adequately-powered studies, and randomized trials and a control for the covariates are needed to support our findings. Therefore, there is the need for more studies that corroborate the potential role of microbiota and intestinal permeability in response to pharmacologic treatment in RA, as well as to validate IFABP2 as a new marker related to lack therapeutic response. 

## 4. Materials and Methods

### 4.1. Studied Population 

A cross-sectional pilot study was conducted in 23 women diagnosed with RA according to the American College of Rheumatology (ACR) and the European League Against Rheumatism (EULAR) criteria [61]. They were diagnosed in the Rheumatology Department of the General Hospital in Chilpancingo, Mexico, from March 2019 to February 2020. Patients with an overlapping syndrome (characteristics of two autoimmune connective tissue diseases present in the same patient); chronic viral infection (hepatitis C or B virus or human immunodeficiency virus); or acute bacterial, virus, or fungal infections were excluded from the study. In addition, RA patients with gastrointestinal surgeries (e.g., gastrectomy, bariatric surgery, and colectomy) or who were diagnosed with peptic ulcer and inflammatory bowel diseases were also excluded, as well as those who had antibiotic treatment two or three months before taking the sample, drug treatment for other condition, laxatives and current extreme diet (e.g., parenteral nutrition or macrobiotic diet), or the consumption of probiotics. All patients were surveyed to obtain sociodemographic data. The clinical and treatment characteristics were evaluated during the consultation and from the clinical file. The weekly average dose and cumulative dose of methotrexate (MTX) and prednisone (PDN) were calculated. The cumulative dose was defined as the sum of all the doses taken by the patient (mg of drugs consumption according to the number of weeks). In contrast, the average dose was defined as the weekly dose between the numbers of appointments. All patients agreed to participate and gave their informed consent in writing in accordance with the Declaration of Helsinki. The protocol was approved by the Ethics and Research Committee of the University Autonomous of Guerrero (project identification code CB-004/2017).

### 4.2. Sample Collection

Blood samples were collected after 8 h of overnight fasting. Serum samples were stored at −20 °C until used. The erythrocyte sedimentation rate (ESR) was determined by the Wintrobe method. The C reactive protein (CRP) and rheumatoid factor (RF) were determined using the immunoturbidimetry technique (Ortho Clinical Diagnostics, VITROS^®^5600, Raritan, NJ, USA). An RF level ≥ 20 UI/mL was considered positive. On the other hand, stool samples were collected in sterile vials and stored at a temperature of −80 °C for subsequent analysis. 

### 4.3. Clinical Assessment

The disease activity and disability were evaluated through the Disease Activity Score 28 (DAS28) and a Spanish version of the Health Assessment Questionnaire-Disability Index (HAQ-DI). The 13 RA patients were treated with conventional synthetic disease-modifying anti-rheumatic drugs (csDMARDs) and corticosteroids (Cs). Meanwhile, ten newly diagnosed patients, defined as new-onset RA (NORA) and untreated, provided their samples previous to starting the consumption of their prescribed treatment. The treated RA patients received an oral MTX to the standard of care doses that was prescribed by their rheumatologist. The clinical response phenotype was defined in the 13 treated RA patients according to the DAS28-ESR change. The phenotypes were defined as following: response, primary non-response, and secondary non-response [62].

### 4.4. DNA Extraction from Stool and qPCR to Estimation of Microbial Abundance

Bacterial DNA was extracted with the QIAamp DNA stool Minikit (Qiagen Inc., Valencia, CA, USA), following the manufacturer’s instructions. DNA concentration and purity were determined with a Nanodrop N1000 (Thermo Fisher Scientific, Carlsbad, CA, USA). The count of gut microbiota was determined with specific primers [63,64,65,66,67] shown in Supplementary Table S1. To determine the microbiota abundance, curves of calibration were performed using *Bacteroides fragilis* ATCC 25285, *Prevotella melanonigenica* ATCC 25845, *Porphyromonas gingivalis* ATCC 33277, and *Escherichia coli* ATCC 25922 reference strains, as well as with the *Lactobacillus* strain of *Lactobacillus casei* Shirota of Yakult^®^. Serial dilutions from bacteria cells ranging between 18 × 10^6^ at 18 × 10^0^ were used to isolate DNA and performed the qPCR curves of calibration. 

The qPCR was performed using 5 μL Maxima SYBR Green/ROX (Thermo Scientific, Vilnius, Lituania), 0.31 μL of specific primers (0.25 μmol/L), and 1 μL of DNA (20 ng) in a total 12.5 μL volume. Negative controls were included in each experiment. The calibration curves of each bacterium were duplicated in each assay. The fluorescence products were detected in the reaction cycles using the equipment StepOnePlus Real-Time PCR System (Applied Biosystems, Foster City, CA, USA). The abundance was quantified using cycle threshold (Ct) values, and the data was graphically represented in Log, base 10 (log 10).

### 4.5. Cytokines (TNF-α, IL-10, and IL-17A) and Serum IFABP2 Levels Quantification 

The TNF-α serum levels were determined using a commercial ELISA kit (Invitrogen, Thermo Fisher Scientific, Vienna, Austria) following the manufacturer’s instructions, and the results were expressed as pg/mL. The IL-10 and IL-17A levels were determined with a ProcartaPlex Hu assay (Invitrogen, Thermo Fisher Scientific, Vienna, Austria), and the results were expressed as pg/mL. Similarly, the IFABP2 serum levels were determined with ELISA (Sigma Aldrich, St. Louis, MO, USA) according to the manufacturer protocol, and the results were expressed as ng/mL. 

### 4.6. Statistical Analysis

Statistical analysis was carried out using Stata v. 13.0 (StataCorp, College Station, TX, USA) and GraphPad Prism v. 8.0 (GraphPad Software, San Diego, CA, USA) for Windows. Continuous parametric data is presented as the mean ± standard deviation (SD) and compared using a *t*-student test. Non-parametric data is presented as median and (5th–95th) percentile and compared using Mann-Whitney U-Tests. Categorical data is summarized, reporting numbers, and proportions (%) and compared using Fisher’s exact test. Spearman’s rank correlation was used to calculate correlation between this study’s dependent and independent variables. *p*-Values below 0.05 were considered statistically significant.

## 5. Conclusions

The increase of intestinal permeability is potentially related to the early changes in gut microbiota’s bacteria. The microbial ratios, based on the abundance of *Lactobacillus* and *Porphyromonas gingivalis*, tend to be related to the secondary non-response to pharmacologic therapy in RA. Therefore, intestinal permeability could establish the preamble for the therapeutic secondary non-response in RA.

## Figures and Tables

**Table 1:** Clinical, inflammatory markers and intestinal bacteria abundance in the group RA patients.

Characteristics	NORA (*n* = 10)	Treated (*n* = 13)	*p*-Value
Clinical assessment			
Age, years ^a^	44.8 ± 10.11	45.69 ± 11.94	0.851
Disease evolution, months ^b^	19.5 (4–192)	60 (12–300)	0.087
ESR, mm/hr ^a^	36.90 ± 10.56	36.15 ± 15.30	0.896
CRP, mg/L^b^	9.5 (4.99–46.9)	20.1 (4.99–33.9)	0.092
DAS28-ESR, score ^a^	4.94 ± 1.56	3.28 ± 0.76	0.002
HAQ-DI, score ^b^	0.57 (0–1.2)	0.10 (0–1)	0.134
Rheumatoid factor, UI/mL ^b^	72 (8.59–984.3)	103.5 (8.59–942.5)	0.619
Positive rheumatoid factor ≥ 20 UI/mL, *n* (%) ^c^	7 (70)	11 (84.62)	0.618
**Inflammatory markers**			
TNF-α, pg/mL ^b^	23.33 (13.33–65.33)	26.66 (17.33–46.66)	0.419
IL-10, pg/mL ^b^	8.64 (0–55.78)	6.36 (0.11–47.55)	0.732
IL-17A, pg/mL ^b^	3.105 (0–48.04)	5.46 (0–34.86)	0.707
IFABP2, ng/mL ^b^	21.18 (7.46–29.16)	11.64 (5.186–28.82)	0.035
**Specific group bacterial**			
*Lactobacillus*, Log_10_ CFU/mL ^b^	6.26 (5.33–9.38)	5.93 (4.32–8.59)	0.192
*Bacteroides fragilis*, Log_10_ CFU/mL ^b^	2.85 (1.38–4.45)	2.86 (0.60–5.14)	0.804
*Prevotella*, Log_10_ CFU/mL ^b^	5.73 (1.72–6.88)	6.35 (2.58–7.02)	0.664
*Porphyromonas gingivalis*, Log_10_ CFU/mL ^b^	−0.56 (−0.80–0.03)	−0.71 (−1.61–0.03)	0.340
*Escherichia coli*, Log_10_ CFU/mL ^b^	3.45 (2.51–4.68)	3.05 (2.07–5.01)	0.385
**Current therapy scheme**			
MTX, *n* (%) ^c^	-	3 (23.08)	
MTX + PDN, *n* (%) ^c^	-	6 (46.15)	
MTX + CLQ + PDN, *n* (%) ^c^	-	4 (30.77)	

^a^ Data are expressed as the mean and standard deviation (±), compared using the *t*-student test. ^b^ Data are expressed as the median and percentiles 5th–95th, compared using the Mann Whitney U test. ^c^ Data are expressed as the *n* (%), compared using the Fisher exact test. *p*-Value < 0.05 was considered statistically significant.

## Data Availability

The data that support the findings of this study are available from the author [I.P.G.-G.], upon reasonable request.

## Supplementary Materials

The following supporting information can be downloaded at: https://www.mdpi.com/article/10.3390/ijms24031958/s1.

## Abbreviations

ACPAs	antibodies to citrullinated protein antigens
ACR	American College of Rheumatology
CD14	cluster of differentiation 14
CFU	colony-forming unit
CLQ	Chloroquine
CRP	C reactive protein
Cs	corticosteroids
CsDMARDs	conventional synthetic disease modifying anti-rheumatic drugs
DAS28	Disease Activity Score 28
DAS28-ESR	Disease Activity Score-28 with Erythrocyte Sedimentation Rate
DNA	deoxyribonucleic acid
ELISA	Enzyme-Linked ImmunoSorbent Assay
ESR	erythrocyte sedimentation rate
EULAR	European League Against Rheumatism
HAQ-DI	Health Assessment Questionnaire-Disability Index
IFABP2	intestinal fatty-acid binding protein 2
IFN-γ	interferon-gamma
IL-6	interleukin-6
IL-10	interleukin-10
IL-17A	interleukin-17A
IL-1β	interleukin-1 beta
IL-22	interleukin-22
IL-23	interleukin-23
LBP	lipopolysaccharide-binding protein
LPS	lipopolysaccharide
MLCK	myosin light chain kinase
MTX	methotrexate
NORA	new-onset RA
PDN	prednisone
qPCR	quantitative polymerase chain reaction
RA	rheumatoid arthritis
RF	rheumatoid factor
sCD14	soluble cluster of differentiation 14
TGF-β	transforming growth factor-β
Th1	T helper 1 cells
Th17	T helper 17 cells
TLR-2	Toll-like receptor 2
TNF-α	tumor necrosis factor alpha
ZO-1	zonula occludens-1
