# Supplementary material for: Serum Levels of IFABP2 and Differences in Lactobacillus and Porphyromonas gingivalis Abundance on Gut Microbiota Are Associated with Poor Therapeutic Response in Rheumatoid Arthritis: A Pilot Study
Source: Int J Mol Sci. 2023 Jan 19;24(3):1958. doi: 10.3390/ijms24031958 (PMC9916456; doi:10.3390/ijms24031958)
Supplement: Supplementary file 1 [file ijms-24-01958-s001.zip › ijms-2090284-supplementary.pdf]

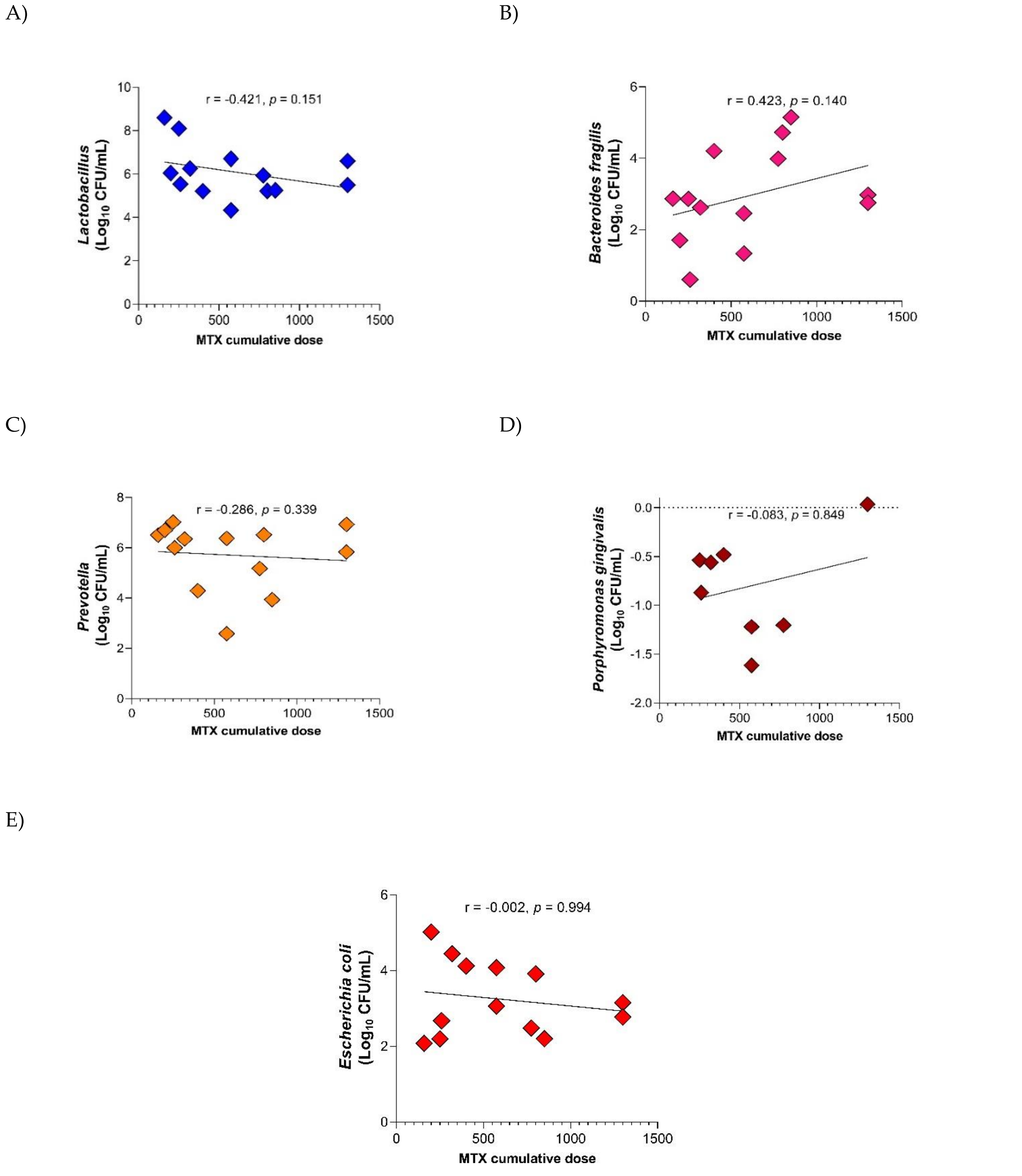

Figure S1. Correlation between MTX cumulative doses with the gut microbiota in RA patients with treatment. (A*)* *Lactobacillus*, (B) *Bacteroides fragilis*, (C) *Prevotella*, (D) *Porphyromonas gingivalis*, and (E) *Escherichia coli*. Spearman's correlation. r=rho, correlation coefficient. *p*-Value <0.05 was considered statistically significant.

A)

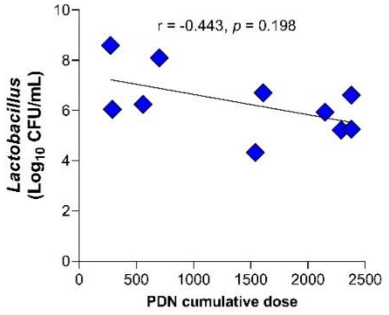

B)

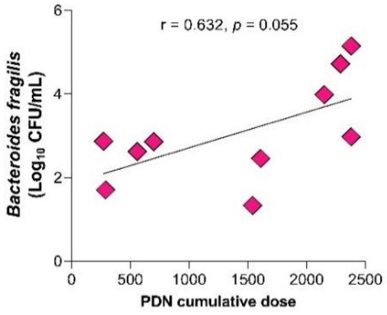

C)

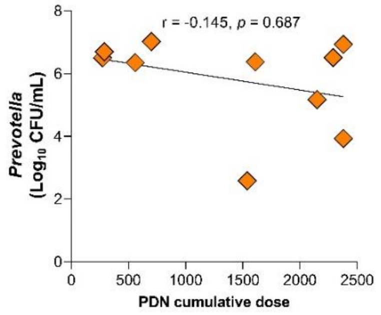

D)

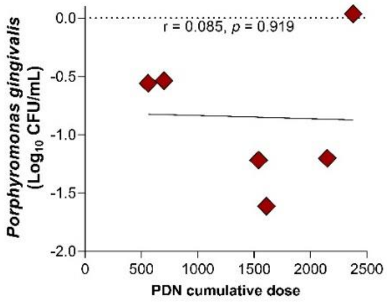

E)

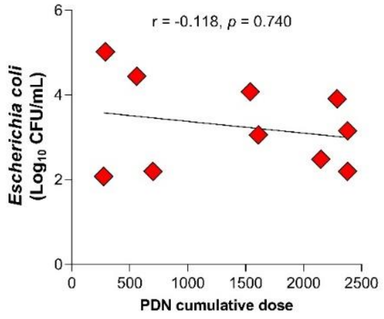

Figure S2. Correlation between PDN cumulative doses with the gut microbiota in RA patients with treatment. (A) *Lactobacillus*, (B) *Bacteroides fragilis*, (C) *Prevotella*, (D) *Porphyromonas gingivalis*, and (E) *Escherichia coli*. Spearman's correlation. r=rho, correlation coefficient. *p*-Value <0.05 was considered statistically significant.

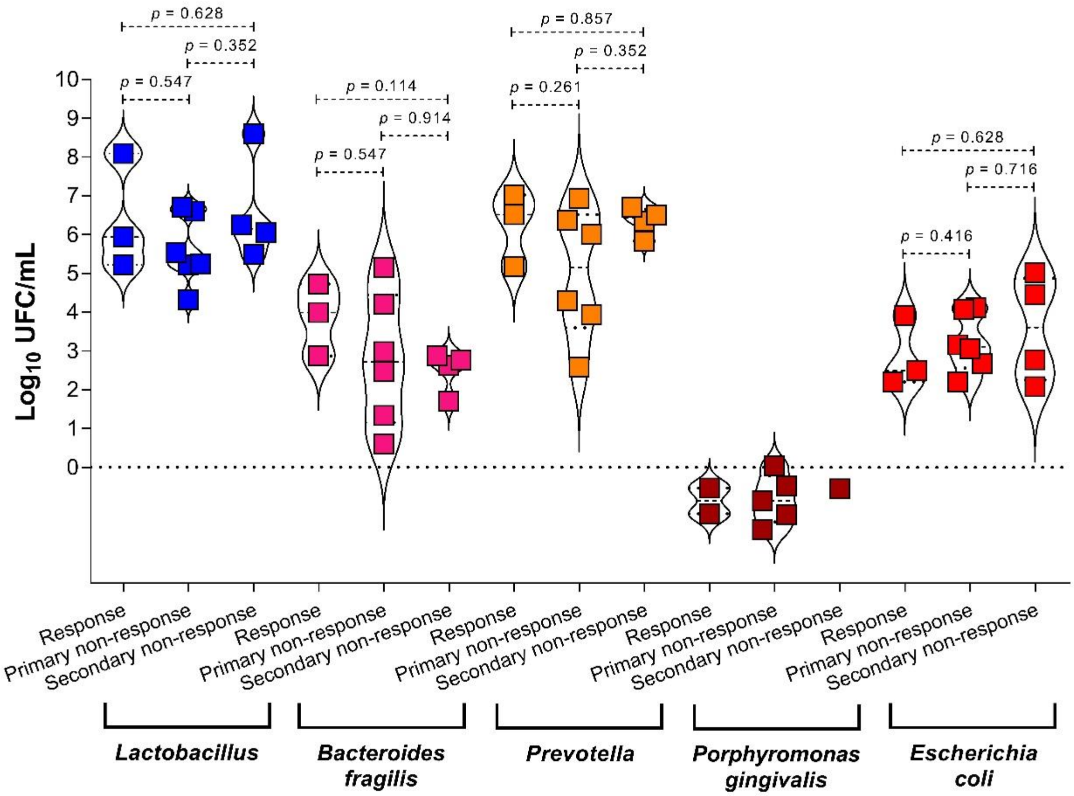

Figure S3. Association of gut microbiota according to response to phenotype to therapy in RA patients. Statistical analyses were performed by Mann Whitney U test. *p*-Value <0.05 was considered statistically significant.

Table S1. Sequences of primers of bacterial specific for qPCR.

Bacterial group	Primer	Sequence	Product size (pb)	Cycles and amplification conditions	Reference
Firmicutes					
Lactobacillus	F	CGATGAGTGCTAGGTGTTGGA	186	40 cycles	[63]
	R	CAAGATGTCAAGACCTGGTAAG		5 min at 94°C, 20 s at 94°C, 20 s at 55°C, 50 s at 72°C, 30 s at 94°C	
Bacteroidetes					
Bacteroides fragilis	g-Bfra-F	ATAGCCTTTCGAAAGRAAGAT	495	40 cycles	[64,65]
	g-Bfra-R	CCAGTATCAACTGCAATTTTA		5 min at 94°C, 20 s at 94°C, 20 s at 50°C, 30 s at 72°C, 1 min at 72°C	
Prevotella	g-Prevo-F	CACRGTAAACGATGGATGCC	513	40 cycles	[64,65]
	g-Prevo-R	GGTCGGGTTGCAGACC		5 min at 94°C, 20 s at 94°C, 20 s at 58.7°C, 50 s at 72°C, 1 min at 72°C	
Porphyromonas gingivalis	F	AGGCAGCTTGCCATACTGCG	404	40 cycles	[66]
	R	ACTGTTAGCAACTACCGATGT		2 min at 94°C, 20 s at 94°C, 20 s at 60°C, 20 s at 72°C, 1 min at 72°C	
Proteobacteria					
Escherichia coli	F	CATGCCGCGTGTATGAAGAA	95	40 cycles	[67]
	R	CGGGTAACGTCAATGAGCAAA		5 min at 94°C, 20 s at 94°C, 20 s at 60°C, 50 s at 72°C, 1 min at 72°C	

qPCR, quantitative polymerase chain reaction.
